# Respiratory symptoms and illnesses related to the concentration of airborne particulate matter among brick kiln workers in Kathmandu valley, Nepal

**DOI:** 10.1186/s40557-017-0165-0

**Published:** 2017-03-27

**Authors:** Seshananda Sanjel, Sanjay N. Khanal, Steven M. Thygerson, William S. Carter, James D. Johnston, Sunil K. Joshi

**Affiliations:** 1 Department of Community Medicine, Kathmandu University School of Medical Sciences, Dhulikhel Hospital, Dhulikhel, Kavre Nepal; 2 Environment Science, Department of Environment Science and Engineering, School of Science, Kathmandu University, Dhulikhel, Nepal; 3 Environmental and Occupational Health, Department of Health Science, Brigham Young University, Provo, UT USA; 4 Environmental Management, University of Findlay, Findlay, OH USA; 5 Department of Community Medicine, Kathmandu Medical College, Kathmandu University, Kathmandu, Nepal

**Keywords:** Suspended particulate matter, Similar exposure group, Brick kiln, Respiratory symptoms/illnesses, Nepal

## Abstract

**Background:**

Bricks have been manufactured in Nepal for hundreds of years and are seen as a component of Nepalese sculpture and architecture. Large quantities of hazardous materials including high concentrations of particulate matter are emitted on a daily basis from brick kilns. Exposure to these hazardous materials can lead to adverse consequences on the environment and human health. This study was conducted to  estimate the prevalence of respiratory symptoms/illnesses and the magnitude of respirable and total dust exposures among Nepalese brick kiln workers.

**Methods:**

Respiratory symptoms/illnesses were evaluated by questionnaire among brickfield workers (*n* = 400) and a referent group of grocery workers (*n* = 400) in Kathmandu valley. Work zones (WZs): green brick molding (GBM), green brick stacking/carrying (GBS/C), red brick loading/carrying (RBL/C), coal preparation (CP) and firemen (FM) were the similar exposure groups (SEGs) from where personal air samples and interviews were taken. Among brickfield workers, personal monitoring was conducted across SEGs for total (*n* = 89) and respirable (*n* = 72) dust during February–March 2015 and March–April 2016. Applying multi-stage probability proportionate to size sampling technique, 16 kilns and 400 brick workers for interview were selected. Proportions, means, medians and ranges were calculated for the demographics, samples and respiratory symptoms/illnesses. One-way ANOVA was applied to compare the significance differences of the level of particulate matter among SEGs. Bivariate and multivariate logistic regression analysis were performed to evaluate association between respiratory symptoms/illnesses and participants groups, and SEGs among brick kiln workers at 0.05 level. Statistical analyses were performed using IBM SPSS Statistics 21.

**Results:**

Chronic cough (14.3%), phlegm (16.6%) and bronchitis (19.0%) were higher (*P* < 0.05) among brickfield compared with grocery workers (6.8, 5.8 and 10.8%). Mean respirable (5.888 mg/m^3^) and total (20.657 mg/m^3^) dust exposures were highest for red brick loading tasks. The prevalence of chronic cough, chronic phlegm, chronic bronchitis, wheezing and asthma were significantly higher for other WZs workers (*p* < 0.05) compared with CP; for GBM: 22.9, 34.6, 15.0 and 7.5%; for GBS/C: 13.5, 15.8, 10.0, 8.8 and 7.5%; for RBL/C: 11.1, 17.1, 27.4, 19.0 and 11.9%; for FM: 18.4, 12.5, 28.4, 4.9 and 0.0%; and for CP: 4.9, 6.3, 13.3, 9.3 and 4.0% respectively.

**Conclusion:**

High dust exposures identified in this study may explain the increased prevalence of respiratory symptoms/illnesses among Nepalese brickfield workers, warranting action to reduce exposures.

## Background

Brick manufacturing, particularly by hand molding, dates back thousands of years in Nepal, and continues to be a major industry in the Kathmandu valley and other areas throughout the country [1–3]. Currently and historically, bricks have been one the most common structural building materials used in the Kathmandu valley [3–5]. Furthermore, the Kathmandu valley has experienced significant population growth since the 1950s, creating a demand for more bricks and brick kilns [6]. As of 2000, there were over 100 kilns in operation in the valley, employing an estimated 30,000 seasonal workers [3, 7, 8].

Brick manufacturing has been identified as a major source of ambient air pollution in the Kathmandu valley. Current manufacturing processes include construction of buildings, construction of roads, and brick kilning. Operation time lasts from November until the middle of May when the brick kilns are in full production. In each case, bio-fuels, coal, and used tires are used as fuel sources in brick kilns [9]. Accordingly, several studies show positive associations between ambient air pollution levels and seasonal operation of brick kilns in Kathmandu valley [2, 10–12]. Similar findings have been reported in Budgam, India, and in Bangladesh [13–15]. Fatima [13] reported ambient air pollution concentrations of total suspended particulates (TSP) and respirable suspended particulates (RSP) as ^3^12.0 and 123.0 μg/m^3^ and 25.0 and 194 μg/m^3^, during the operational and non- operational seasons of brick kiln, respectively in Budgam, India. A study in Bangladesh revealed that brick kiln emissions contributed 7 to 99 μg/m^3^ to urban air pollution levels during brick kiln operation [15]. A study carried out during pre-operation, operation and post-operation phases in India uncovered that the RSP levels were 0.216, 0.241 and 0.214 mg/m^3^, respectively [16]. In Kathmandu valley, differences in ambient TSP between the operating and non-operating brick kiln seasons were 56.0 and 33.0 μg/m^3^, respectively, and for PM_10_ were 50 and 29 μg/m^3^, respectively [11]. Similarly, in another study, the average TSP during the off-season was 0.265 mg/m3 whereas it reached to 0.634 mg/m^3^ during operation time [12]. Another study in Nepal shows, the PM_10_ concentration in an area with brick kilns was found to be 0.569 mg/m^3^, while it was only 0.158 mg/m^3^ in an area without brick kilns [12]. Among sources, the brick industry contributes one third of all the dust pollution in the Kathmandu Valley [12, 17].

Although several studies have evaluated ambient air pollution associated with brick kilns, few studies report results of occupational exposures among brick kiln workers. Brick kiln workers are closer to pollution sources and likely experience higher exposures than the general public. Using direct-reading area sampling, Raza et al. [18] found PM_10_ concentrations in Pakistani brick kilns were 888, 1830, and 861 μg/m^3^ for molding and loading, firing, and unloading sections, respectively, while PM_2.5_ concentrations were 301, 307, and 628 μg/m^3^ for modulation and loading, burning, and unloading sections, respectively. Myers et al. [19] reported mean total and respirable dust levels of 15.16 and 2.22 mg/m^3^, respectively, among South African brick workers. Breathing zone dust levels varied considerably by job category; however, traditional brick kilns used in the Kathmandu valley may have significantly different exposure profiles. Occupational exposures in brick kilns may be one or more orders of magnitude greater than ambient levels, suggesting brick kiln workers are at greater risk of respiratory-related diseases.

Studies clearly show that brickfield workers and people in the surrounding community are more likely to suffer from illnesses caused by the kilns’ pollution [11, 20]. In addition, workers engaged in different work tasks inside brick kilns suffered variety of respiratory illnesses [2, 21–23]. In a study comparing brickfield workers to non-brickfield workers, 42% of brickfield workers had respiratory illnesses compared to just 9.3% for the referent group of workers [21]. A later study showed that illnesses such as chronic cough and phlegm, chest tightness were significantly higher among brickfield workers compared to referent workers [22, 23]. Respiratory illnesses reported in previous studies are thought to result from a variety of particulate exposures encountered in brick manufacturing. However, there has been little attention given to differential particulate exposures based on similar exposure groups (SEGs).

The objective of this study, therefore, was to evaluate the prevalence of respiratory symptoms and illnesses, and to estimate the magnitude of particulate exposures according to SEGs among brickfield workers.

## Methods

A cross-sectional study was conducted in Kathmandu valley that includes three densely populated districts (Kathmandu, Lalitpur and Bhaktpur) targeting brick kiln workers. There were 106 operating brick kilns in Kathmandu Valley at the time of sampling and interviews. Among them, 62 brick kilns were in Bhaktpur, 26 brick kilns were in Lalitpur and 18 brick kilns were in Kathmandu district. First, all the brick kilns were visited and added as part of the potential sampling frame.

Multi-stage probability proportionate to size (PPS) sampling was applied to select brick kilns and brickfields workers [24]. In total, 9 kilns from Bhaktapur, 4 kilns from Lalitpur and 3 kilns from Kathmandu district were selected. A total of 800 participants, 400 brick workers as exposed and 400 grocery workers from Bhaktapur, Lalitpur, Kathmandu districts proportionately as referent group were recruited for the interviews. The aim of this study was to to find out the additional contribution of exposure to the brick kiln for the workers as there is already air pollution in the valley with significant contribution of respiratory health [25]. The groceries and grocery worker were taken as referent group. This group was chosen as the reference group because both the groups were from the similar socioeconomic background. Brick kilns workers who had been working for more than 1 year were included in the study. Workers who had been working for more than 1 year in small and middle groceries, excluding large shopping malls and roadside huts were interviewed. Field surveys were completed during February–March 2015 using structured interviews administered by trained health workers making unannounced visits to brick kilns.

A strategy has been developed based on grouping workers who have similar job duties within a production unit of a plant. This processing of grouping is called similar exposed groups (SEGs) [26, 27]. Brickfield SEGs are commonly classified as green brick molding (GBM), green brick stacking/carrying (GRS/C), red brick loading/carrying (RBL/C), coal preparation (CP) and fireman (FM) zones. It stands to reason that different job classifications may result in widely different exposures, both in concentration of contaminant, and in the constituents that make up the particles.

Socio-demographic characteristics and health data were assessed applying a Nepali version of the field pre-tested questionnaire. Monitoring and supervision of interviewers was done by the principal investigator frequently during interview time in the field.

Personal air samples were collected following NIOSH Method 0500 for TSP and NIOSH Method 0600 for RSP. During the course of the survey, 89 personal samples for TSP and 72 personal samples for RSP were obtained using SKC AirChek® 52 personal air sampling pumps pre-calibrated to a flow rate of 2.0 liters per minute for TSP and 2.5 liters per minute for RSP. Average sampling time was 120 min for TSP and 160 min for RSP. Pre- and post-calibration was completed to verify flow rates using a DryCal® Defender 510 primary volumetric flow standard. For TSP, particulate air samples were collected on pre-weighed 5.0 *u*m PVC membrane filters (37 mm) placed in-line with pump air flow, and clipped to the collar within the employees’ breathing zone. For RSP, particulate air samples were collected on pre-weighed 5.0 *u*m PVC membrane filters (37 mm) with aluminum cyclone all placed in-line with pump air flow, and clipped to the collar within the employees’ breathing zone. The samples, including field and laboratory blanks, were analyzed using gravimetric methods at ALS Laboratories in Salt Lake City, Utah, USA.

It was assumed that the exposure for the various workers would be the same for the entire workday of 8 h. Workers did not transfer to other jobs during the day. Thus, before interpreting the results all the values were converted to 8 h time weighted average (TWA) using the following formula: TWA = [(C_1_ × T_1_) + (C_2_ × T_2_) + (C_n_ × T_n_]/8 h; where C = concentration for T_n_ (mg/m^3^), T = sampling time (hours).

Proportions, mean, median and range were calculated for socio-demographics (age, gender, marital status, schooling and duration of work) and particulate matter (TSP, RSP). The prevalence with 95% confidence intervals (CIs) for all respiratory symptoms and illnesses (chronic cough, chronic phlegm, chronic bronchitis, wheezing and asthma) within a year were estimated. One way ANOVA analysis was applied to compare the level of RSP and TSP among work zones (WZs) using log_10_ transferred PM values as the values were skewed. Bivariate and multivariate logistic regression analysis was carried out to evaluate associations between respiratory symptoms/illnesses and participant groups (exposed and referent) and SEGs among brickfield workers. CP zone workers were used as the reference group in the logistic regression analysis among SEGs because the exposure to coal dust was viewed as a much different hazard compared to the brick dust to which the other SEGs were exposed. For instance, coal dust exposure may be a greater risk to workers in coal crushing, while respirable silica may be the primary particulate exposure during brick molding. The level of significance was set at <0.05 level. Statistical analyses were performed using the IBM SPSS Statistics 21, Armonk, NY, USA. To calculate 95% CI of the prevalence, stat calculator online software was used.

Ethical approval for study was obtained from the institutional review committee of Kathmandu University School of Medical Sciences (IRC-KUSMS). Participation in the study was voluntary and written consent was obtained from the brick kiln owners before obtaining any data. Written consent (thumb print in case of illiterate interviewees) to publish the data was obtained from each interviewee before interviews.

Operational definitions of the study outcomes [22, 28]:

Chronic Cough: cough as much as 4–6 times per day occurring for most days of the week (≥5 days) for at least 3 months of the year and for at least two consecutive years.

Chronic Phlegm: sputum expectoration as much as twice a day for most days of the week (≥5 days) for at least 3 months of the year and for at least two consecutive years.

Chronic Bronchitis: cough and sputum expectoration occurring for most days of the week (≥5 days) for at least 3 months of the year and for at least two consecutive years.

Wheezing: chest ever sounds wheezy or whistling most days or nights in the past 2 months

Asthma: at least two or more attacks of shortness of breath with wheezing (whistling sound on expiration) in the past 2 months with normal breathing in between episodes of shortness of breath or diagnosed asthmatic by a physician.

Ever smoker: more than 20 packs of cigarettes in a lifetime or more than 1 cigarette a day for 1 year.

Never smoker: less than 20 packs of cigarettes in a lifetime or less than 1 cigarette a day in 1 year.

## Results

**Fig. 1:**
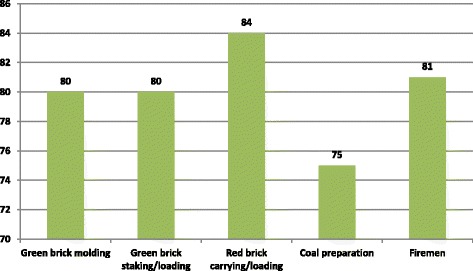
Frequency of brick kiln workers among SEGs (*n* = 400)

The questionnaire was completed by 800 participants [exposed: 400 (50%); referent: 400 (50%)]. Almost 20.0% of exposed workers were less than 19 years of age whereas only three percent of referent were included in this age group. The mean ± SD age of exposed was 31.74 ± 12.97 years with range of 12 to 73 years and for referent was 33.33 ± 9.03 years with range of 14 to 68 years. Among those, 63.0% of brick workers achieved only primary education whereas almost 95% of grocery workers attained secondary and university education. Almost 40.0% among exposed and 35.0% among referent were ever smokers whereas 84.0% of exposed and 85.7% of referent were current smokers (Table 1). Among workers, 20.0% workers were from GBM and GBS/S each, 21.0% were from RBL/C, 18.8% were from CP, and 20.2% of them were FM zones (Fig. 1).

**Table 1:** Socio-demographics of participants

Socio-economic variables	Response groups			
	Exposed		Referent	
	Frequency	%	Frequency	%
Age group of the respondents				
≤ 19 years	81	20.2	12	3.0
20–29 years	119	29.8	129	32.2
30–39 years	84	21.0	166	41.5
40–49 years	68	17.0	72	18.0
50–59 years	33	8.2	16	4.0
60–69 years	11	2.8	5	1.2
≥ 70 years	4	1.0	0	0
Total	**400**	**100.0**	**400**	**100.0**
Exposed: mean age = 31.74 years, SD of age = 12.97 years, range of age = 12 to 73 years Control: mean age = 33.33 years, SD of age = 9.03 years, range of age = 14 to 68 years				
Gender				
Female	102	25.5	130	32.5
Male	298	74.5	270	67.5
Total	**400**	**100.0**	**400**	**100.0**
Attainment of formal education				
No	238	59.5	30	7.5
Yes	162	40.5	370	92.5
Total	**400**	**100.0**	**400**	**100.0**
Levels of education				
Primary	102	63.0	16	4.3
Lower secondary	43	26.5	76	20.5
Secondary and higher secondary	14	8.6	203	54.9
University	3	1.9	75	20.3
Total	**162**	**100.0**	**370**	**100.0**
Duration of work in years				
≤ 5 years	265	66.2	237	59.2
6–10 years	63	15.8	113	28.2
11–15 years	30	7.5	28	7.0
16–20 years	23	5.8	19	4.8
≥ 21 years	19	4.8	3	0.8
Total	**400**	**100.0**	**400**	**100.0**
Ever smoker				
No	238	59.5	260	65.0
Yes	162	40.5	140	35.0
Total	**400**	**100.0**	**400**	**100.0**
Current smoker				
No	26	16.0	20	14.3
Yes	136	84.0	120	85.7
Total	**162**	**100.0**	**140**	**100.0**

The mean concentrations of RSP were 0.722, 1.877, 5.888, 2.454 and 1.133 mg/m^3^ respectively for GBM, GBS/C, RBL/C, CP and MF zone workers respectively. Similarly, the mean concentrations of TSP were 4.581, 1.671, 16.271, 20.657, 7.094 and 3.435 mg/m^3^ respectively for GBM, GBC, GBS, RBL/C, CP and FM zone workers respectively (Table 2).

**Table 2:** Concentration of suspended particulate matters (mg/m^3^)

Respirable suspended particulate (RSP) mg/m^3^						
Parameters	Green brick molding (*n* = 6)	Green brick carrying (*n* = 0)	Green brick stacking (*n* = 17)	Red brick loading (*n* = 16)	Coal preparation (*n* = 14)	Fireman (*n* = 19)
Mean	0.722	-	1.877	5.888	2.454	1.133
Median	0.590	-	2.000	4.150	0.990	0.610
SD	0.554	-	0.961	5.040	2.753	1.402
Minimum	0.270	-	0.380	0.470	0.050	0.120
Maximum	1.800	-	3.800	16.000	7.900	5.800
Total suspended particulate (TSP) mg/m^3^						
Parameters	Green brick molding (*n* = 9)	Green brick carrying (*n* = 5)	Green brick stacking (*n* = 17)	Red brick loading (*n* = 21)	Coal preparation (*n* = 17)	Fireman (*n* = 20)
Mean	4.581	1.671	16.271	20.657	7.094	3.435
Median	1.663	1.750	12.250	23.373	2.888	1.181
SD	7.405	1.578	14.952	12.492	8.38750	8.814
Minimum	0.100	0.100	0.110	1.840	0.510	0.270
Maximum	23.370	3.680	42.000	49.880	23.370	40.250

The relationship of the extent of PM concentration was assessed among SEGs using ANOVA. For RSP the relationship was significant with *F* value of 6.632 (*P* < 0.001) and for TSP, the relationship was significant with *F* value of 10.184 (*p* < 0.000). When performed multiple comparisons applying Tukey’s test to assess the extent of RSP among SEGs, the relationship was significant for GBM and RBL/C (*p*: 0.007). Likewise, RBL/C and CP (*p*: 0.012) and FM (*p*: <0.001) also showed high significance for the extent of RSP. Similarly, the TSP concentrations among SEGs for GBM and RBL/C (*p*: 0.002) had significant association. Alike, the red brick loading zone had a significant relationship and GBS/C (*p*: 0.018), CP (*p*: 0.010) and FM (*p*: <0.001). Also, fire masters zone was significant with green brick stacking (*p*: 0.002).

**Fig. 2:**
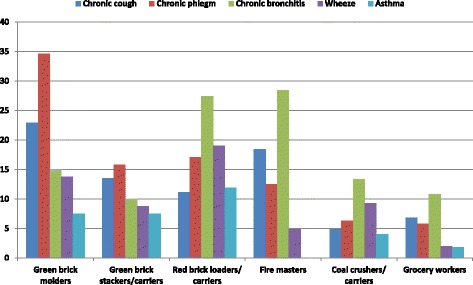
Prevalence of respiratory illnesses among SEGs (%)

Almost 55% of exposed and 29% of referent reported coughing. among those who complained of cough, 29.5% of exposed and 12.8% of referent usually cough as much as 4 to 6 times a day, 4 or more days out of the week. Thus, the prevalence of chronic cough was 14.3% (9.91 to 18.69%) for exposed and 6.8% (2.92 to 10.68%) for referent populations. Nearly 42% of exposed and 11% of referent produced phlegm; among total participants 8.0% exposed and 3.0% referent usually produced phlegm as much as 4 to 6 times a day, 4 or more days out of the week. Consequently, the prevalence of chronic phlegm was 16.6% (10.99 to 22.21%) for exposed and 5.8% (0.86 to 10.74%) for referents. Approximately 25% of exposed and 16.0% of referents had episodes of cough and phlegm; amongst workers expectorating cough and phlegm, 19.0% (15.16 to 22.84%) of exposed and 10.8% (7.76 to 13.84%) of referent endured chronic bronchitis. Correspondingly, 11.3% of exposed and just two percent of referent were ill with wheezing and almost six percent of exposed and two percent of referent reported having asthma (Table 3). The prevalence of chronic cough, chronic phlegm, chronic bronchitis, wheezing and asthma among SEGs were as follow: GBM: 22.9%, 34.6, 15.0%, 13.8% and 7.5%; GBS/C: 13.5%, 15.8, 10.0%, 8.8% and 7.5%; RBL/C: 11.1, 17.1, 27.4, 19.0 and 11.9%; FM: 18.4%, 12.5%, 28.4%, 4.9% and nil; CP: 4.9, 6.33, 13.3, 9.3 and 4.0% (Fig. 2).

**Table 3:** Chronic respiratory symptoms/illnesses for the response groups

Respiratory illnesses	Exposed		Referent	
	Frequency	%	Frequency	%
Cough				
Absent	179	44.8	285	71.2
Present	221	55.2	115	28.8
Total	**400**	**100.0**	**400**	**100.0**
Usually cough as much as 4 to 6 times a day, 4 or more days out of the week				
Absent	103	25.8	64	16.0
Present	118	29.5	51	12.8
Does not apply	179	44.8	285	71.3
Total	**400**	**100.0**	**400**	**100.0**
Chronic cough				
Absent	209	85.7	151	93.2
Present	35	14.3 (9.91–18.69)	11	6.8 (2.92–10.68)
Total	**244**	**100.0**	**162**	**100.0**
Phlegm				
Absent	231	57.8	355	88.8
Present	169	42.2	45	11.2
Total	**400**	**100.0**	**400**	**100.0**
Usually bring up phlegm as much as 4 to 6 times a day, 4 or more days out of the week				
Absent	137	34.3	74	18.5
Present	32	8.0	12	3.0
Does not apply	231	57.8	314	78.5
Total	400	100.0	400	100.0
Chronic phlegm				
Absent	141	83.4	81	94.2
Present	28	16.6 (10.99–22.21)	5	5.8 (0.86–10.74)
Total	**169**	**100.0**	**86**	**100.0**
Episodes of cough and phlegm				
Absent	302	75.5	336	84.0
Present	98	24.5	64	16.0
Total	**400**	**100.0**	**400**	**100.0**
Chronic episodes of cough and phlegm				
Absent	324	81.0	357	89.3
Present	76	19.0 (15.16–22.84)	43	10.8 (7.76–13.84)
Total	**400**	**100.0**	**400**	**100.0**
Wheezing on most days or nights				
Absent	355	88.8	392	98.0
Present	45	11.3	8	2.0
Total	**400**	**100.0**	**400**	**100.0**
Attack of wheezing cause shortness of breath (asthma)				
Absent	375	93.8	393	98.3
Present	25	6.3	7	1.8
Total	**400**	**100.0**	**400**	**100.0**

Logistic regression analysis was carried out to compare association between participants’ groups and chronic respiratory symptoms adjusted with age, duration of work and smoking practices. Brick industry workers were about two times more likely to have chronic cough (OR: 2.30; 95% CI: 1.13–4.67; *p*: 0.021) compared with grocery workers. The adjusted OR slightly increased and was significant (OR: 2.35; 95% CI: 1.14–4.85; p: 0.021). Age of participants and duration of work were significant predictors (*P* < 0.001 each) for chronic cough. Similarly, brick workers were almost three times more likely to suffer chronic phlegm (OR: 3.22; 95% CI: 1.20–8.66; *p*: 0.021) in comparison to referent participants. When adjusted it was still almost three times more likely (AOR: 2.98; 1.07–8.24; p: 0.036). Duration of work was a significant predictor (*p* < 0.001) for chronic phlegm. Likewise, brick industry workers were approximately two times more likely to have chronic bronchitis (OR: 1.95; 95% CI: 1.30–2.91; *p*: 0.001) when compared with grocery workers. Adjusted OR was also more or less the same (OR: 1.91; 95% CI: 1.26–2.90; *p*: 0.002) indicating age and smoking practice of workers were significant predictors (*p* < 0.001 each) of chronic bronchitis. In the same way, brick workers were six times more likely to have chronic wheezing (OR: 6.21; 95% CI: 2.89–13.36; *p* < 0.001) in comparison to grocery workers when adjusted the AOR remain more or less same (OR: 6.00; 95% CI: 2.78–12.94, *p* < 0.001). Smoking habits of the participants were a significant predictor (*p* < 0.001) for chronic wheezing. Also, exposed participants were about four times more likely to experience asthma (OR: 3.74; 95% CI: 1.60–8.76; *p*: 0.002) compared with referents. On adjustment, AOR slightly decreased but remained significant (OR: 3.18; 95% CI: 1.34–7.58; p: 0.009).

Logistic regression analysis compared the association between chronic respiratory symptoms/illnesses and type of work performed by the brick industry workers. Green brick molders were nearly six times more likely to experience chronic cough (OR: 5.80; 95% CI: 1.20–27.93; *p*: 0.028) when compared with workers involved in coal crushing/carrying work. Age and duration of work were significant predictors for chronic cough (*p*: 0.001 each). There was no association between chronic phlegm and other work types (green brick stacking/carrying, red brick loading/carrying and firing) when compared with coal crushing/carrying work. Upon adjustment the association did not exist.

Green brick molders were about eight times more likely to develop chronic phlegm (OR: 7.94; 1.54–41.10; *p*: 0.013) compared to workers involved in coal crushing/carrying work. The association could not persevere up on adjustment. Duration of work of the participants was a significant predictor (*p* < 0.001) for developing chronic phlegm. There was no association between chronic phlegm and other work types (green brick stacking/carrying, red brick loading/carrying and firing) when compared with coal crushing/carrying work. There was no association when adjusted with the predictors.

Workers involved in red brick loading/carrying were over two times more likely to get chronic bronchitis (OR: 2.45; 95% CI: 1.10–5.57; *p*: 0.032) while compared with workers involved in coal crushing/carrying work. Similarly, fire masters were almost three times more likely of getting chronic bronchitis (OR: 2.58; 95% CI: 1.13–5.87; *p*: 0.024) compared with workers involved in coal crushing/carrying work. Age of workers (*p*: 0.003) and smoking habit of workers (*p*: 0.002) were significant predictors for causing chronic bronchitis. While adjustment of predictors was carried out, workers involved in red brick loading/carrying were approximately three times more likely to have chronic bronchitis (OR: 3.08; 95% CI: 1.26–7.51; *p*: 0.013), but, after adjustment, there was no association between chronic phlegm and other work types (green brick molding, green brick stacking/carrying and firing). Smoking practice of the workers was a significant predictor (*p*: 0.018) of wheezing among work types. Smoking habits of the workers was a significant predictor (*p*: 0.026) for presence of asthma among work types (Table 4).

**Table 4:** Regression analysis between respiratory symptoms/illnesses adjusted with age, duration of work and smoking

Symptoms/illnesses	OR (95% C.I.)	AOR (95% C.I.)
Participants group		
Chronic cough		
Exposed	2.30 (1.13–4.67)^a^	2.35 (1.14–4.85)^a^
Referent	Reference	Reference
Chronic phlegm		
Exposed	3.22 (1.20–8.66)^a^	2.98 (1.07–8.24)^a^
Referent	Reference	Reference
Episodes of chronic cough and phlegm		
Exposed	1.95 (1.30–2.91)^b^	1.91 (1.26–2.90)^b^
Referent	Reference	Reference
Chronic wheeze		
Exposed	6.21 (2.89–13.36)^c^	6.00 (2.78–12.94)^c^
Referent	Reference	Reference
Asthma		
Exposed	3.74 (1.60–8.76)^b^	3.18 (1.34–7.58)^b^
Referent	Reference	Reference
Similar exposure group		
Chronic cough		
Green brick molding	5.80 (1.20–27.93)^a^	4.46 (0.84–23.75)
Green brick stacking/carrying	3.03 (0.60–15.40)	5.61 (0.99–31.78)
Red brick loading/carrying	2.44 (0.45–12.76)	4.13 0.71–24.09)
Firing	4.39 (0.89–21.61)	2.24 (0.41–12.17)
Coal crushing/carrying	Reference	Reference
Chronic phlegm		
Green brick molding	7.94 (1.54–41.10)^a^	2.79 (0.43–18.17)
Green brick stacking/carrying	2.81 (0.53–15.03)	4.43 (0.72–27.39)
Red brick loading/carrying	3.01 (0.60–16.02)	3.95 (0.66–23.62)
Firing	2.14 (0.36–12.63)	0.66 (0.09–5.13)
Coal crushing/carrying	Reference	Reference
Chronic bronchitis		
Green brick molding	1.15 (0.46–2.84)	1.15 (0.44–2.99)
Green brick stacking/carrying	0.72 (0.27–1.94)	0.94 (0.33–2.68)
Red brick loading/carrying	2.45 (1.10–5.57)^a^	3.08 (1.26–7.51)^a^
Firing	2.58 (1.13–5.87)^a^	2.03 (0.84–4.87)
Coal crushing/carrying	Reference	Reference
Chronic wheezing		
Green brick molding	1.55 (0.57–4.23)	1.35 (0.48–3.82)
Green brick stacking/carrying	0.93 (0.31–2.80)	0.90 (0.29–2.81)
Red brick loading/carrying	2.29 (0.88–5.91)	2.12 (0.79–5.67)
Firing	0.51 (0.14–1.80)	0.39 0.11–1.47)
Coal crushing/carrying	Reference	Reference
Asthma		
Green brick molding	1.95 (0.47–8.10)	1.75 (0.40–7.63)
Green brick stacking/carrying	1.95 (0.47–8.10)	1.95 (0.45–8.52)
Red brick loading/carrying	3.24 (0.87–12.30)	3.06 (0.77–12.08)
Firing	0	0
Coal crushing/carrying	Reference	Reference

^a^Significant at 0.05 level

^b^Significant at 0.01 level

^c^Significant at 0.001 level

## Discussion

This study involved 800 participants (400 exposed and 400 referent populations). Socio-demographic results of this study reveal significant differences between these two industries. Specifically, compared with grocery stores, brick kilns were more likely to employ children under the age of 18 and older workers above the age of 60. There are fewer females working in the brick kilns which may be due to the greater hazard found in the kilns. Workers in brick kilns have less education but tend to stay working in the brick kiln for a longer duration. Additionally, smoking was more prevalent among workers in the brick kilns.

### Illnesses

In our current study the prevalence of chronic cough, chronic phlegm and chronic bronchitis, wheeze and asthma matched with a similar study conducted in Egypt which studied the same symptoms among brick kiln workers and those in retail sales [29]. Similar findings also are seen for respiratory illnesses based on studies in Pakistan, Egypt and India [22, 23, 30]. This study’s findings are also supported by a study conducted in Nepal indicating that there was greater health burden for communities and school children who live near brick kilns [11] and research conducted in Ethiopia showed cement factory workers had significantly higher prevalence of respiratory symptoms compared with Civil Servants [24]. Studies in similar industries to brick making, such as the heavy clay in Iran and the leather industry in Pakistan, also show similar health effects from particulate matter exposures [29, 31, 32]. Nepalese brick workers generally have no provision of pre-employment and periodic medical examination or health insurance, thus they are predisposed to the development of multiple respiratory diseases [33].

Logistic regression analysis was carried out to compare associations between respondents group and chronic respiratory symptoms adjusted with age, duration of work and smoking status. In our current study, respiratory symptoms: chronic cough, chronic phlegm, chronic bronchitis, wheeze, asthma, dyspnea, and chest tightness had significant higher prevalence for exposed workers compared with referent workers. These findings were consistent with a study conducted in Croatia [23].

Our current study revealed that the ages of participants and duration of work were significant predictors of chronic cough; duration of work was a significant predictor for chronic phlegm; Age of workers and smoking were significant predictors for chronic bronchitis; and smoking was a significant predictor for chronic wheeze. All the above respiratory symptoms were significant at <0.001 level of confidence. These outcomes are similar to prior studies from Eygpt and Croatia showing that workers engaged in the brick manufacturing industry are at risk of developing respiratory impairment, and the degree of this impairment is directly correlated with the duration of the service [34], age of workers, duration of work, and smoking habit [23]. The Egyptian study showed that brick kiln workers had a significantly higher frequency of chronic respiratory problems compared to their referent group and the most common chronic respiratory symptoms among them were chronic cough followed by dyspnea, chest wheeze, chronic bronchitis, and asthma indicating the duration of work and smoking habits [29].

The Tanzanian study conducted in cement factory revealed that exposed workers had more chronic cough, chronic sputum production, dyspnea, work-related shortness of breath and chronic bronchitis in comparison to referents and longer duration to dust exposure [35]. Another study carried out in Ethiopia also revealed that the prevalence of respiratory symptoms were significantly higher among cement factory exposed workers, in which age of workers and the smoking practice were the significant predictors at <0.05 level of confidence [24].

A study carried out in lather industry in Pakistan revealed that the prevalence of asthma appeared to be high and is associated with educational status, smoking and duration of work [32].

Logistic regression analysis was carried to compare association between chronic respiratory symptoms and SEGs in the brick kiln workers. Green brick molders had significantly higher prevalence of chronic cough compared to coal crushers/carriers. Age and duration of work were significant predictors for chronic cough at <0.01 level of confidence. Green brick molders experienced significantly high prevalence of chronic phlegm compared to workers involved in coal crushing/carrying work for which duration of work of the participants was a significant predictor at 0.001 level of confidence. Workers involved in red brick loading/carrying and firing had significantly higher prevalence of chronic bronchitis compared with workers involved in coal crushing/carrying work both in crude and adjusted conditions. Age of workers (*p* < 0.01) and smoking habit of workers (*p* < 0.01) were significant predictors for causing chronic bronchitis. Smoking practice of the workers was a significant predictor (*p* < 0.05) of wheezing among work types. Smoking habit of the workers was a significant predictor (*p* < 0.05) for presence of asthma among work types. In a previous study Sheikh et al., found that workers involved in brick baking were about four times more likely to have both chronic bronchitis and asthma compared to those involved in carriage and placement work [22]. In the study conducted in Egypt, a significant high prevalence of respiratory problems was found among brick kiln workers especially the brick bakers [29].

### Exposure to dust particles for brick kiln workers

In our current study, the mean and maximum exposures to RSP were 2.588 and 16.00 mg/m^3^, and to TSP were 12.190 and 57.000 mg/m^3^. Among SEGs, red brick loading had the significantly higher mean and maximum values RSP (mean: 5.888 mg/m^3^ and maximum: 16.000 mg/m^3^) and TSP (mean: 20.657 mg/m^3^ and maximum: 49.880 mg/m^3^) in comparison to TLV of ACGIH (RSP: 3 mg/m^3^ and TSP: 10 mg/m^3^) [36]. Our findings are in general agreement with Myers et al. [19], who found 2.22 and 15.16 mg/m^3^ for RSP and TSP among brick workers, respectively.

Particulate exposures among brick kiln workers are known to vary considerably based on job categories [19]. In our study, when breathing zone exposures were assessed by SEGs we found that workers in green brick molding and red brick loading had significantly different exposures to RSP. This finding was not surprising. In green brick molding, brick materials are wet, which would allow for less suspension of dust particles. Conversely, red brick loading exposes workers to airborne particulates from smoke and from dry dust from fired bricks. In terms of occupational exposure, a study in Nepal compared the average value of PM_10_ and TSP for pre-operation time of brick kilns (0.029 and 0.033 mg/m^3^) and kiln operation time (0.050 and 0.056 mg/m^3^), and concluded that ambient air pollution in rural areas due to brick kilns is a genuine problem [11]. No previous study in brick kiln with assessment of RSP and TSP along with health conditions of workers was found. From our current study, it can be concluded that SEGs which had significantly high extent of PM concentrations had significantly more respiratory symptoms and illnesses.

### Implication in Nepal

There is a remarkably high level of RSP and TSP in brickfields. A significant high prevalence of respiratory problems was found among brick kiln workers. To improve the work environment, this study will be valuable for developing the preventive and protective approaches to the brick industry sector under supervision of Ministry of Industry and Labor. It will also support technology transfer, engineering controls and enforcement for using personal protective equipments by promulgating laws.

## Conclusion

While several prior studies show significantly greater respiratory symptoms/illnesses among brick kiln workers compared to referent groups, there is little data on the influence that job category has on exposure or respiratory symptoms/illness. Based on our findings, workers engaged in red brick loading and green brick stacking tasks experienced higher particulate exposures compared with workers in other job categories. Red brick loaders suffered disproportionately from wheezing, asthma and chronic bronchitis compared to other SEGs. These results suggest protective measures are needed, particularly among work groups with higher exposures or higher prevalence of respiratory symptoms/illness. In addition, these results suggest future research is needed to identify particulate matter constituents and how they vary by job classification. For instance, green brick molders were among the lowest RSP and TSP exposures, but they experienced the highest prevalence of chronic cough and phlegm.

There is a resounding need for improved evaluation of the hazards within the kilns in the Valley and the risks posed to the workers and the community. Control areas of focus in the brick kilns include: improve the quality fuel used in the kilns; update kiln technology; crackdown on illegal kilns in the Valley; decrease the long hours on the job leading to greater risk of overexposure; and provide personal protective equipment (PPE) to the workers. No governmental standards on occupational exposure have been promulgated yet. A reactive legislation approach to control is falling behind, with occupational safety and health standards unenforced; nevertheless, priorities geared towards the health of the community and the workers in the brick kilns are increasingly pertinent with the rise in industry.

Voluntary organizational efforts to promote and educate the workers in the kilns, their employers and the general public on the necessity of industrial hygiene practice should be considered. These measures to investigate further into the industrial hazards of the brick kilns in Kathmandu Valley, Nepal and mitigation strategies will help promulgate the need for greater intervention. All workers deserve a workplace environment free from extreme health hazards and unsafe conditions.

## Abbreviation

ACGIH: American Conference of Governmental Industrial Hygienists
ANOVA: Analysis of variance
AOR: Adjusted odds ratio
CI: Confidence interval
CP: Coal preparation
FBI: Federation of Nepal brick Industry
FM: Firemen
GBM: Green brick molding
GBS/C: Green brick stacking/carrying
IRC-KUSMS: Institutional review committee of Kathmandu University School of Medical Science
NIOSH: National Institute of Occupational Safety and Health
OR: Odds ratio
PM: Particulate matters
PPS: Probability proportionate to size
PVC: Polyvinyl chloride
RBL/C: Red brick loading/carrying
RSP: Respirable suspended particulate
SD: Standard deviation
SEG: Similar exposure group
TSP: Total suspended particulate
TWA: Time weighted average
WZs: Work zones

## References

[1] GEFONT (2007). Nepal: Labour Under the Chimney–A Study on the Brick Kilns of Nepal.

[2] Pariyar SK, Das T, Ferdous T (2013). Environment And Health Impact For Brick Kilns In Kathmandu Valley. Int J Sci Technol Res.

[3] Haack BN, Khatiwada G (2007). Rice and bricks: environmental issues and mapping of the unusual crop rotation pattern in the Kathmandu Valley, Nepal. Environ Manag.

[4] Bajracharya SB (2015). The Thermal Performance of Traditional Residential Buildings in Kathmandu Valley. J Inst Eng.

[5] Chaulagain H, Rodrigues H, Silva V, Spacone E, Varum H. Earthquake loss estimation for the Kathmandu Valley. Bulletin of Earthquake Engineering. 2016;14(1):59-88.

[6] Segupta U, Bhattarai Upadhyay V (2016). Lost in transition? Emerging forms of residential architecture in Kathmandu. Cities.

[7] Giri D, Murthy KV, Adhikary PR, Khanal SN. Ambient air quality of Kathmandu valley as reflected by atmospheric particulate matter concentrations (PM10). International Journal of Environmental Science & Technology. 2006;3(4):403-10.

[8] Environmental and Public Health Organization (ENPHO) (2001). A study on status of brick industry in the Kathmandu Valley.

[9] Maskey Manandhar U, Dangol SB (2013). Study on Evaluating Energy Conservation Potential of Brick Production in SAARC Countries: A Report on Nepal.

[10] Jha PK, Lekhak HD (2003). Air Pollution Studies and Management Efforts in Nepal. Pure Appl Geophys.

[11] Joshi SK, Dudani I. Environmental health effects of brick kilns in Kathmandu valley. Kathmandu Univ Med J. 2008;6(21):3–11. 18604107

[12] Raut AK (2003). Brick Kilns in Kathmandu Valley: Current status, environmental impacts and future options. Himal J Sci.

[13] Fatima I (2011). Impact of Brick Kiln Emissions on the Ambient Air Quality and Vegetation: A Case Study of District Budgam.

[14] Darain KM, Rahman ABM, Ahsan A, Islam ABMS, Yusuf B (2013). Brick Making Practice in Bangladesh: A Review of Energy Efficiency and Air Pollution Scenarios. J Hydro Environ Res.

[15] Guttikunda SK, Begum BA, Wadud Z. Particulate pollution from brick kiln clusters in the Greater Dhaka region, Bangladesh. Air Qual Atmos Health. 2012.

[16] Hussan A, Bhat GA, Sheikh MA (2013). Impact of Brick Kiln and Vehicular Emissions on Lichen Diversity in Khanabal Area of Anantnag District (J&K), India. Int Res J Environ Sci.

[17] Ghimire H (2008). An Assessment of the Environmental Problems in the Kathmandu Valley of Nepal.

[18] Raza A, Qamer MF, Afsheen S, Adnan M, Naeem S, Atiq M (2014). Particulate Matter Associated Lung Function Decline in Brick Kiln Workers of Jalalpur Jattan, Pakistan. Pak J Zool.

[19] Myers JE, Lewis P, Hofmeyr W (1989). Respiratory health of brickworkers in Cape Town, South Africa. Scand J Work Environ Health.

[20] Banibrata Das B (2014). Assessment of Occupational Health Problems and Physiological Stress among the Brick Field Workers of West Bengal, India. Int J Occup Med Environ Health.

[21] Joshi SK (2013). Occupational Health and Safety Assessment of Child Workers in the Brick Industry, Nepal.

[22] Shaikh S, Nafees AA, Khetpal V, Jamali AA, Arain AM, Yousuf A (2012). Respiratory symptoms and illnesses among brick kiln workers: a cross sectional study from rural districts of Pakistan. BMC Public Health.

[23] Zuskin E, Mustajbegovic J, Schachter EN, Kern J, Doko-Jelinic J, Godnic-Cvar J (1998). Respiratory Findings in Workers Employed in the Brick-Manufacturing Industry. J Occup Environ Med.

[24] Turner AG. Sampling strategies: Handbook on Designing of Household Sample Surveys. Geneva: United Nations Secretariat: Statistics Division; 2003.

[25] Karki KB, Dhakal P, Shrestha SL, Joshi HD, Aryal KK, Poudyal A (2016). Situation Analysis of Ambient Air Pollution and Respiratory Effects in Kathmandu Valley.

[26] Ghasemkhani M, Kumashiro M, Rezaei M, Anvari AR, Mazloumi A, Sadeghipour HR (2006). Prevalence of Respiratory Symptoms among Workers in Industries of South Tehran, Iran. Ind Health.

[27] Spear JE. Industrial Hygiene Exposure Assessments: Worst-Case Versus Random Sampling. 19314 Timber Ridge Drive, Suite 100 Magnolia, Texas 77355: J.E. Spear Consulting, LLC; 2004. Available from: http://www.jespear.com/articles/04-07-worstcase-vs-random.pdf.

[28] American Thoracic Society. Recommended respiratory disease questionnaires for use with adults and children in epidemiological research. Am Rev Respir Dis. 1978;118:7-53.

[29] Sheta S, El Laithy N (2015). Brick Kiln Industry and Workers’ Chronic Respiratory Health Problems in Mit Ghamr District, Dakahlia Governorate. Egypt J Occup Med.

[30] Monga V, Singh LP, Bhardwaj A, Singh H (2012). Respiratory Health in Brick Kiln Workers. Int J Phys Soc Sci.

[31] Love RG, Waclawski ER, Maclaren WM, Wetherill GZ, Groat SK, Porteous RH (1999). Risks of respiratory disease in the heavy clay industry. Occup Environ Med.

[32] Shahzad K, Akhtar S, Mahmud S (2006). Prevalence and determinants of asthma in adult male leather tannery workers in Karachi, Pakistan: A cross sectional study. BMC Public Health.

[33] WorldBank, URBAIR (1997). Urban Air Quality Management Strategy in Asia: Kathmandu Valley.

[34] Al-Shamma YMH, Dinana FM, Dosh BA (2009). Physiological study of the effect of employment in old brick factories on the lung function of their employees. J Environ Stud.

[35] Rushton L (2007). Review of Literature on Chronic Obstructive Pulmonary Disease and Occupational Exposure to Silica.

[36] American Conference of Governmental Industrial Hygienists. Threshold limit values for chemical substances and physical agents and biological exposure indices: Cincinnati. American Conference of Governmental Industrial Hygienists: OH; 2004.

